# The carbohydrate-binding domain of overexpressed STBD1 is important for its stability and protein–protein interactions

**DOI:** 10.1042/BSR20140053

**Published:** 2014-07-01

**Authors:** Yuanqi Zhu, Mei Zhang, Amber Renee Kelly, Alan Cheng

**Affiliations:** *Department of Biochemistry and Molecular Biology, University of Louisville School of Medicine, 319 Abraham Flexner Way, HSC-A 715, Louisville, Kentucky 40202, U.S.A.

**Keywords:** CBM20, endoplasmic reticulum, glucose, glycogen, STBD1, ubiquitination, Atg8, autophagy-related gene 8, BafA1, bafilomycin A1, CAP, catabolite gene activator protein, CBM20, family 20 carbohydrate binding module, CHX, cycloheximide, ER, endoplasmic reticulum, FBS, fetal bovine serum, GABARAP, γ-aminobutyric acid receptor-associated protein, GABARAPL1, GABARAP-like 1, GBE1, glycogen branching enzyme 1, GDE, glycogen debranching enzyme, GFP, green fluorescent protein, GS, glycogen synthase, HA, haemagglutinin, HEK-293T, human embryonic kidney 293T, HSP, heat-shock protein, NEM, *N*-ethylmaleimide, PTG, protein targeting to glycogen, STBD1, starch-binding domain-containing protein 1, Ub, ubiquitin, WT, wild-type

## Abstract

STBD1 (starch-binding domain-containing protein 1) belongs to the CBM20 (family 20 carbohydrate binding module) group of proteins, and is implicated in glycogen metabolism and autophagy. However, very little is known about its regulation or interacting partners. Here, we show that the CBM20 of STBD1 is crucial for its stability and ability to interact with glycogen-associated proteins. Mutation of a conserved tryptophan residue (W293) in this domain abolished the ability of STBD1 to bind to the carbohydrate amylose. Compared with the WT (wild-type) protein, this mutant exhibited rapid degradation that was rescued upon inhibition of the proteasome. Furthermore, STBD1 undergoes ubiquitination when expressed in COS cells, and requires the N-terminus for this process. In contrast, inhibition of autophagy did not significantly affect protein stability. In overexpression experiments, we discovered that STBD1 interacts with several glycogen-associated proteins, such as GS (glycogen synthase), GDE (glycogen debranching enzyme) and Laforin. Importantly, the W293 mutant of STBD1 was unable to do so, suggesting an additional role for the CBM20 domain in protein–protein interactions. In HepG2 hepatoma cells, overexpressed STBD1 could associate with endogenous GS. This binding increased during glycogenolysis, suggesting that glycogen is not required to bridge this interaction. Taken together, our results have uncovered new insights into the regulation and binding partners of STBD1.

## Abstract 2

STBD1 is a protein with a carbohydrate-binding domain that is implicated in autophagy and glycogen metabolism. Here we show the carbohydrate-binding domain is crucial for its stability and ability to bind to several glycogen-associated proteins.

## INTRODUCTION

STBD1 (starch-binding domain-containing protein 1; also known as Genethonin-1 and GENX-3414) was originally isolated from a human skeletal muscle library [1]. It is predominately expressed in liver, muscle and heart tissues. STBD1 contains an N-terminal 24 amino acid hydrophobic stretch that allows it to associate with a cellular membrane structure, possibly belonging to the ER (endoplasmic reticulum) or lysosomal organelles [2]. At the C-terminal end, the protein contains a CBM20 (family 20 carbohydrate-binding module) [3,4]. Indeed, STBD1 can bind to glycogen *in vitro* [2]. CBM20 and other related domains involved in carbohydrate metabolism are extensively described by the CAZy (carbohydrate-active enzymes) database at http://www.cazy.org/ [5].

Previous histological studies revealed a co-localization of STBD1 with internal muscle membranes [1]. Interestingly, GS (glycogen synthase) possesses a similar localization pattern [6]. In mice with a targeted deletion of GS in muscle or liver, the loss of glycogen levels correlated with the loss of STBD1 levels in those tissues [2]. It has also been shown that fasting increases both STBD1 and glycogen levels in female but not male mice [7]. Conversely, it was shown that STBD1 and glycogen levels are increased in cardiomyocytes exposed to insulin and high (30 mM) glucose conditions [8]. Despite its implication in glycogen metabolism, studies have not delineated its role in this process.

Very little is known about the regulation and binding partners of STBD1. A yeast-two hybrid screen previously identified GABARAP (γ-aminobutyric acid receptor-associated protein) and GABARAPL1 (GABARAP-like 1) as interactors of STBD1 [2]. These two proteins belong to the Atg8 (autophagy-related gene 8) family [9]. Therefore it has been suggested that STBD1 could participate in autophagy and lysosomal mediated glycogen breakdown (glycophagy). In Pompe disease, glycogen abnormally accumulates due to a deficiency in the lysosomal enzyme, acid-α-glucosidase [10]. Interestingly, STBD1 levels are elevated in a mouse model of Pompe disease [11]. However, suppression of STBD1 levels did not affect lysosomal glycogen accumulation. Therefore much remains to be understood concerning its function.

We are particularly interested in the CBM20 of STBD1. Previous work has demonstrated that carbohydrate-binding domains could possess functions in addition to binding glycogen. In a recent study, we noted that the glycogen-binding domain of the GDE (glycogen debranching enzyme) is well conserved across species, from humans to bacteria [12,13]. In particular, there is an invariant stretch of amino acids (YHxGxxWxW) whereby the mutation of the conserved glycine is associated with glycogen storage disease type III in humans [14,15]. We discovered that mutation of this residue, or disruption of this region in the glycogen-binding domain causes proteasomal mediated targeting, as well as inactivation of its enzymatic activities [12,13].

In humans, the CBM20 family of proteins includes Laforin, a dual specificity phosphatase [16,17]. Of interest, mutations in Laforin have been associated with the abnormal accumulation of glycogen in Lafora disease [18]. This includes a W32G mutation in the CBM20 of Laforin [19]. Interestingly, cellular experiments have shown that mutation of this residue can disrupt the activity of Laforin and its ability to dimerize [20], although other studies indicate that Laforin exists primarily in a monomeric form [21].

Given this information, we hypothesized that the CBM20 of STBD1 possesses additional, undiscovered functions. In this study, we revealed that, in addition to being crucial for binding to carbohydrates, the CBM20 of STBD1 also plays a role in protein stability and interaction with several other glycogen-associated proteins. This new information will provide insight into further understanding the cellular role of STBD1.

## MATERIALS AND METHODS

### Chemicals and antibodies

Antibodies in this study were purchased from the following companies: HA (haemagglutinin) and Myc (Santa Cruz Biotechnology); FLAG (Sigma); GS, Hsp70 (heat-shock proteins 70) and Hsp90 (Cell Signaling Technology); GDE (Abgent); and Laforin (Abnova). The proteasomal inhibitor MG132 was purchased from EMD Biosciences. NEM (*N*-ethylmaleimide) was obtained from Sigma.

### Cell culture

COS, HEK-293T (human embryonic kidney 293T), Hela and HepG2 cells were obtained from the A.T.C.C. and grown in DMEM (Dulbecco's modified Eagle's medium; Invitrogen) containing 10% (v/v) FBS (Invitrogen). All cells were grown in the presence of antibiotics (penicillin and streptomycin) in a humidified chamber with an environment of 5% (v/v) CO_2_.

Stable cell lines were generated using a lentiviral system as previously described with minor modifications [22]. Virus was packaged and produced in HEK-293T cells. Viral supernatants were collected after 48 h, filtered through 0.45 μM filters, and concentrated using Centricon devices (Millipore). Target cells were infected with viral aliquots in the presence of polybrene overnight. Media was changed the next day and the percentage of infected cells was monitored by immunocytochemistry after an additional 24 h. Only cell populations with greater than 80% infection were used.

### Plasmids

Constructs for ubiquitin, Laforin, GDE, PTG (protein targeting to glycogen) and CAP (catabolite gene activator protein) were described previously [12,23,24]. The cDNA for STBD1 was obtained by RT–PCR (reverse transcription–PCR), using RNA isolated from HepG2 cells. All point mutants were conversions to a glycine residue. The NDel mutant was constructed as previously described [2]. STBD1 WT and mutants were cloned into various mammalian expression vectors by PCR. All constructs were verified by sequencing.

### Preparation of cell lysates and immunoblotting

For isolation of lysates for immunoprecipitation, cells were washed in ice-cold PBS and then scraped in lysis buffer [25 mM Tris at pH 7.5, 150 mM NaCl, 1% (v/v) Triton X-100, Complete EDTA-free protease inhibitor]. The lysates were rocked end-over-end for 10 min at 4°C and then clarified by centrifugation at 14000 ***g*** for 10 min. Cleared lysates were subjected to immunoprecipitation with the appropriate antibody and Protein A/G agarose beads (Santa Cruz Biotechnology) for 2 h rotating at 4°C. Immunoprecipitates were extensively washed in lysis buffer and proteins were eluted at 95°C in SDS-loading buffer, separated by SDS/PAGE, and transferred to nitrocellulose. The membranes were processed for Western blotting coupled with ECL (enhanced chemiluminescence) onto film.

For denaturing immunoprecipitations, cells were lysed in denaturing buffer (25 mM Tris at pH 7.5, 150 mM NaCl, 1% Triton X-100, 1% (w/v) SDS, 5 mM NEM, 10 μM MG132) and boiled for 10 min. Lysates were diluted 1:10 with the same buffer lacking SDS and incubated with the appropriate antibody/protein A/G agarose beads for 2 h rotating at 4°C. Immunoprecipitates were extensively washed in lysis buffer (without SDS), and proteins were eluted at 95°C in SDS loading buffer, separated by SDS/PAGE, and transferred to nitrocellulose.

### Confocal fluorescence microscopy

Immunofluorescence studies were performed as previously described [24]. Cells were grown on glass coverslips in 6-well dishes. Following the fixation with 10% (v/v) formalin for 20 min, cells were permeabilized with 0.5% Triton X-100 for 5 min and then blocked with 1% (w/v) BSA and 2% (v/v) goat serum for 1 h. Coverslips were incubated with primary antibodies and Alexa-Fluor conjugated secondary antibodies (Invitrogen) in blocking solution, and mounted on glass slides with Vectashield (Vector Laboratories). Nuclei were stained with DAPI (4′,6-diamidino-2-phenylindole) or TOPRO-3 (Invitrogen). Cells were imaged using confocal fluorescence microscope (Olympus IX SLA). Images were then imported into Photoshop (Adobe Systems, Inc.) for processing.

### GST pull-down assay

GST–STBD1 fusion proteins were expressed in the *Escherichia coli* strain BL21 (Stratagene) and purified as described previously [25]. Mouse liver tissue was excised and lysed as described above for immunoprecipitation. Lysates were incubated with either GST alone or with GST–STBD1 proteins immobilized on glutathione–sepharose beads (Amersham) for 90 min at 4°C. The beads were washed three times with lysis buffer, and the bound proteins were eluted in SDS sample buffer and analysed by Western blotting.

## RESULTS

### Structure and mutants of STBD1

**Figure 1:**
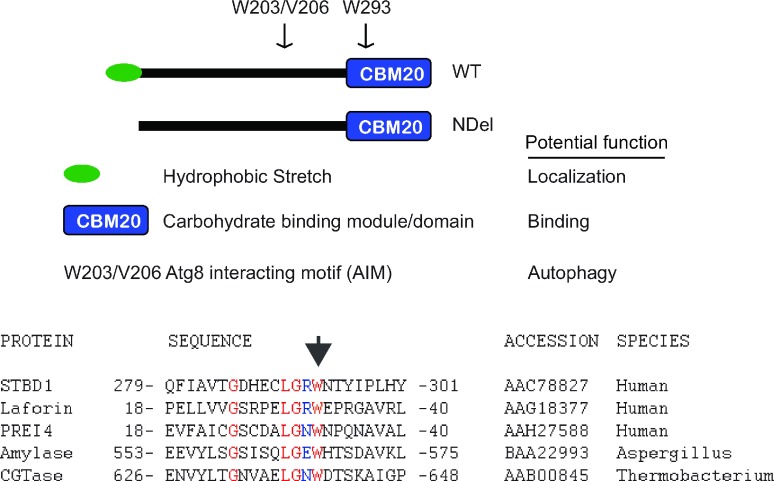
Schematic of STBD1, showing N-terminal hydrophobic domain, C-terminal carbohydrate binding module (CBM20), and key residues within the CBM20 and AIM (Atg8 family interacting motif) NDel denotes the N-terminal deletion mutant (top panel). Sequence alignment of the CBM20 from several family members. Arrow indicates conserved tryptophan that is mutated in Laforin, affecting Lafora disease (bottom panel).

For this study, we generated multiple mutants of STBD1 (Figure 1). These specific mutants were originally described elsewhere [2,26]. Alignment of the CBM20 of STBD1 with others in the family of CBM20 proteins indicates a region of significant identity (shown in red), considering the variety of species in the analysis. We mutated W293 of STBD1, corresponding to the W32 mutant in Laforin. Other point mutants include W203 and V206 (and their combination) which are crucial for binding to GABARAP and GABARAPL1 [26]. Finally, we deleted the first 24 amino acids of STBD1 to remove the hydrophobic stretch (NDel mutant).

### Stability and carbohydrate binding

**Figure 2:**
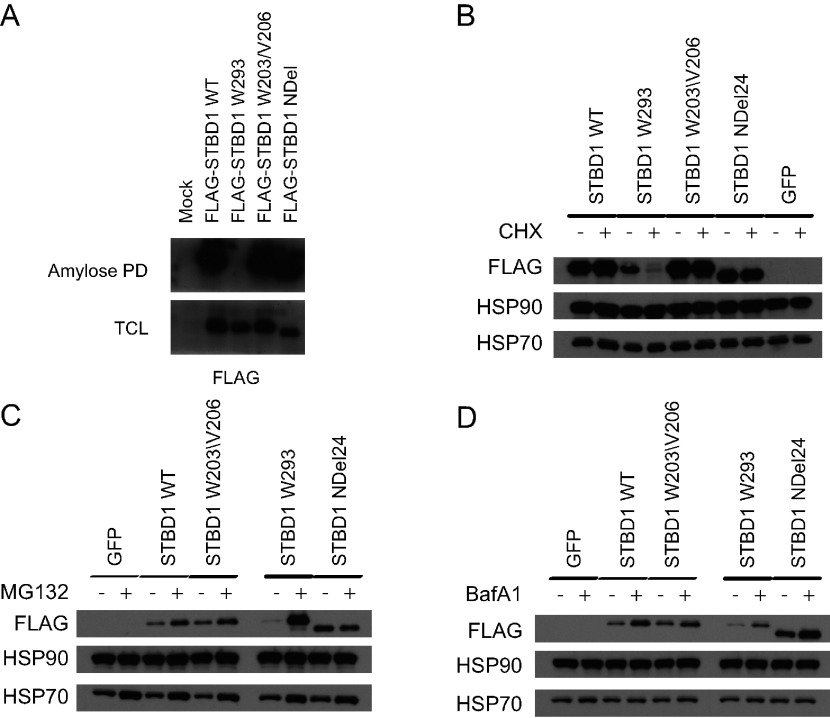
The CBM20 of STBD1 is important for protein stability (**A**) COS cells were transfected with the indicated constructs and subjected to an amylose pull-down assay. All STBD1 proteins bind to amylose except the W293 mutant. (**B**) Hela cells stably expressing the indicated FLAG-tagged STBD1 proteins were left untreated or treated with CHX for 3 h. Lysates were obtained and Western blot analysis performed with the indicated antibodies. (**C**) The Hela cell lines were left untreated or treated with 10 μM MG132 overnight followed Western blot analysis with the indicated antibodies. (**D**) The Hela cell lines were left untreated or treated with 400 nM BafA1 overnight followed Western blot analysis with the indicated antibodies.

We previously demonstrated that mutations in the carbohydrate-binding domain of the GDE disrupted its ability to bind to glycogen and caused it to be targeted for proteasomal degradation [12]. To assess whether the CBM20 of STBD1 might play a similar role, we expressed FLAG-tagged WT and mutant STBD1 proteins in cells, and subjected them to pull-down assays using amylose resin to evaluate carbohydrate-binding (Figure 2A). All STBD1 proteins were able to bind to amylose, except the W293 mutant, confirming that the CBM20 indeed has the ability to bind to carbohydrates.

To determine if these mutants might affect the stability of STBD1, we generated stable Hela cell lines of STBD1 proteins or GFP (green fluorescent protein) as a control. Cells were left untreated or treated with the inhibitor of protein synthesis, CHX (cycloheximide), for 3 h. Protein lysates were obtained and Western blot analysis was performed. Treatment with CHX had no effect on WT, W203/V206 or the NDel mutants, suggesting that their protein half-lives were greater than 3 h (Figure 2B). In contrast, the W293 mutant was almost completely gone after treatment, indicating rapid degradation. As controls, neither Hsp90 or Hsp70 were affected by the treatment.

Proteins that have short half-lives are typically degraded by the proteasome [27]. Indeed, inhibition of the proteasome with MG132 dramatically enhanced the levels of the W293 mutant, while having little effect on the other STBD1 proteins (Figure 2C). Since STBD1 has also been implicated in autophagy, we also tested whether this process could play a role in the degradation of STBD1. Treatment of the cell lines with the autophagy inhibitor, BafA1 (bafilomycin A1) had very little effect on levels of STBD1 (Figure 2D), suggesting that the proteasome plays a more significant role.

### The N-terminus of STBD1 is important for its ubiquitination

**Figure 3:**
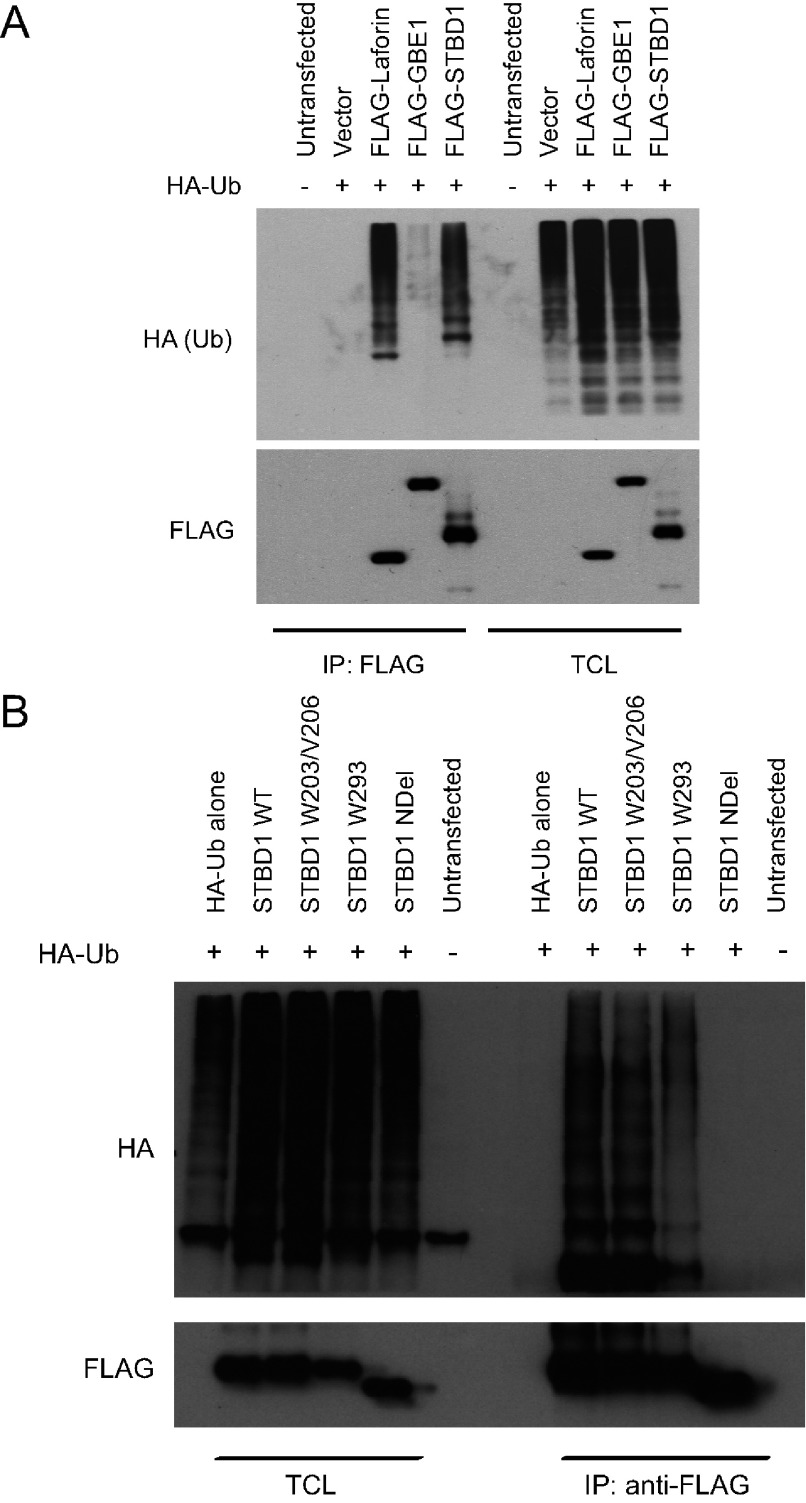
Ubiquitination of STBD1 requires the N-terminal hydrophobic stretch (**A**) COS cells were transfected with the indicated FLAG-tagged constructs along with HA-Ub. Denaturing lysates were obtained and immunoprecipitations with anti-FLAG antibodies were performed, and analysed by Western blotting. (**B**) The same experiment was performed using the indicated STBD1 mutants.

Given that the W293 mutant of STBD1 can be targeted for proteasomal-mediated degradation, we asked whether STBD1 is ubiquitinated. We transfected cells with FLAG-tagged STBD1 along with HA-Ub (HA-tagged ubiquitin). Immunoprecipitations with an anti-FLAG antibody were then performed on denatured lysates followed by Western blotting with an anti-HA antibody (Figure 3A). As controls we also transfected FLAG-tagged Laforin and the GBE1 (glycogen branching enzyme 1). As expected, Laforin immunoprecipitates showed ubiquitination as previously described [23]. Similarly, STBD1 was also ubiquitinated. In contrast, GBE1 was not.

We next assessed whether STBD1 mutants were also ubiquitinated (Figure 3B). Interestingly, STBD1W203/V206 and W293 mutants also displayed ubiquitination, suggesting that an additional signal is required to target the W293 mutant to the proteasome. Moreover, we also discovered that the NDel mutant was not ubiquitinated, suggesting that the N-terminus is required for this process.

### STBD1 interacts with several glycogen-associated proteins

**Figure 4:**
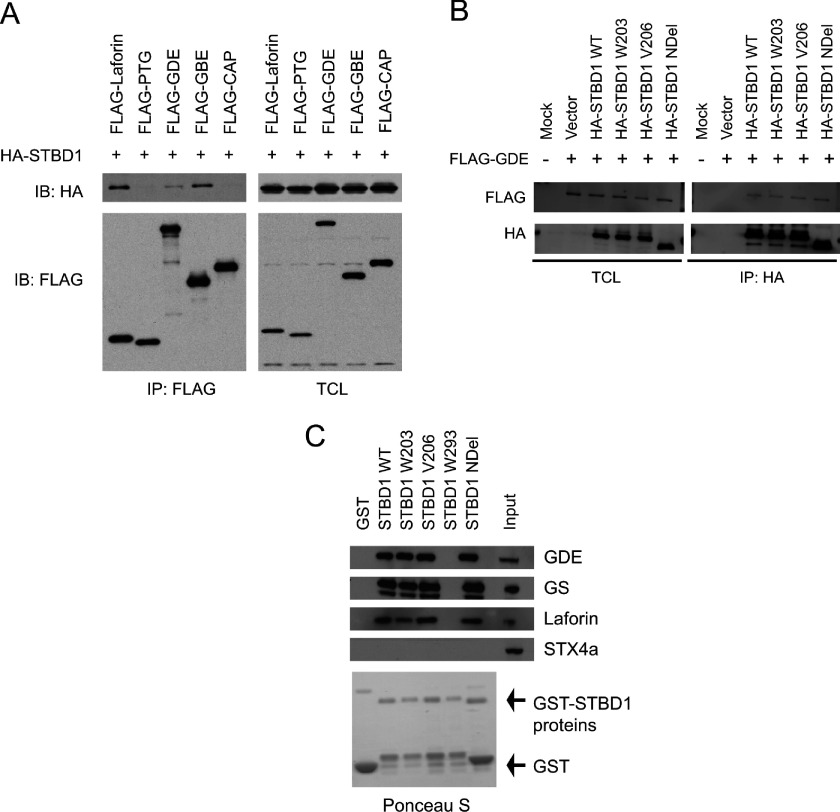
STBD1 interacts with several glycogen-associated proteins (**A**) COS cells were transfected with HA-STBD1 and the indicated FLAG-tagged constructs. FLAG immunoprecipitates were analysed by Western blotting to identify co-precipitating proteins. (**B**) COS cells were transfected with FLAG-tagged GDE and HA-tagged STBD1 constructs. Anti-HA immunoprecipitates were analysed by Western blotting with the indicated antibodies. (**C**) GST alone or GST–STBD1 proteins were produced in *E. coli* and coupled to glutathione–sepharose beads. Mouse liver lysates were then added and pull downs were analysed by Western blotting.

Laforin has previously been shown to interact with several glycogen-associated proteins, such as GS, PTG, GDE, as well as with itself [28,29]. Therefore we asked whether STBD1 could behave similarly. When expressed in cells, co-immunoprecipitation experiments demonstrated that HA–STBD1 could bind to FLAG-tagged Laforin, GBE1 and GDE (Figure 4A). In contrast, neither PTG nor an irrelevant protein (CAP) was able to do so. This suggests that STBD1 does not indiscriminately bind to every glycogen-associated protein.

To test whether different STBD1 mutants would affect the binding, HA-tagged STBD1 proteins were co-transfected with FLAG-tagged GDE. Surprisingly, we found that all the STBD1 mutants we tested were still able to bind to GDE (Figure 4B) and Laforin (results not shown). In these experiments, it was difficult to test the binding to the W293 mutant based on poor expression due to rapid degradation. To overcome this problem, we produced and purified GST-tagged STBD1 proteins in *E. coli*, and performed *in vitro* pull down assays with mouse liver lysates. As shown in Figure 4(C), STBD1 WT, or the W203, V206 and NDel mutants could all bind to endogenous GDE, Laforin and GS. As a control, STX4a (syntaxin 4a) did not. Importantly, mutation of W293 completely abolished binding to the glycogen-associated proteins. These results uncover a novel role for the CBM20 of STBD1 in binding to other proteins.

### Expression of STBD1 causes the relocalization of glycogen-associated proteins

**Figure 5:**
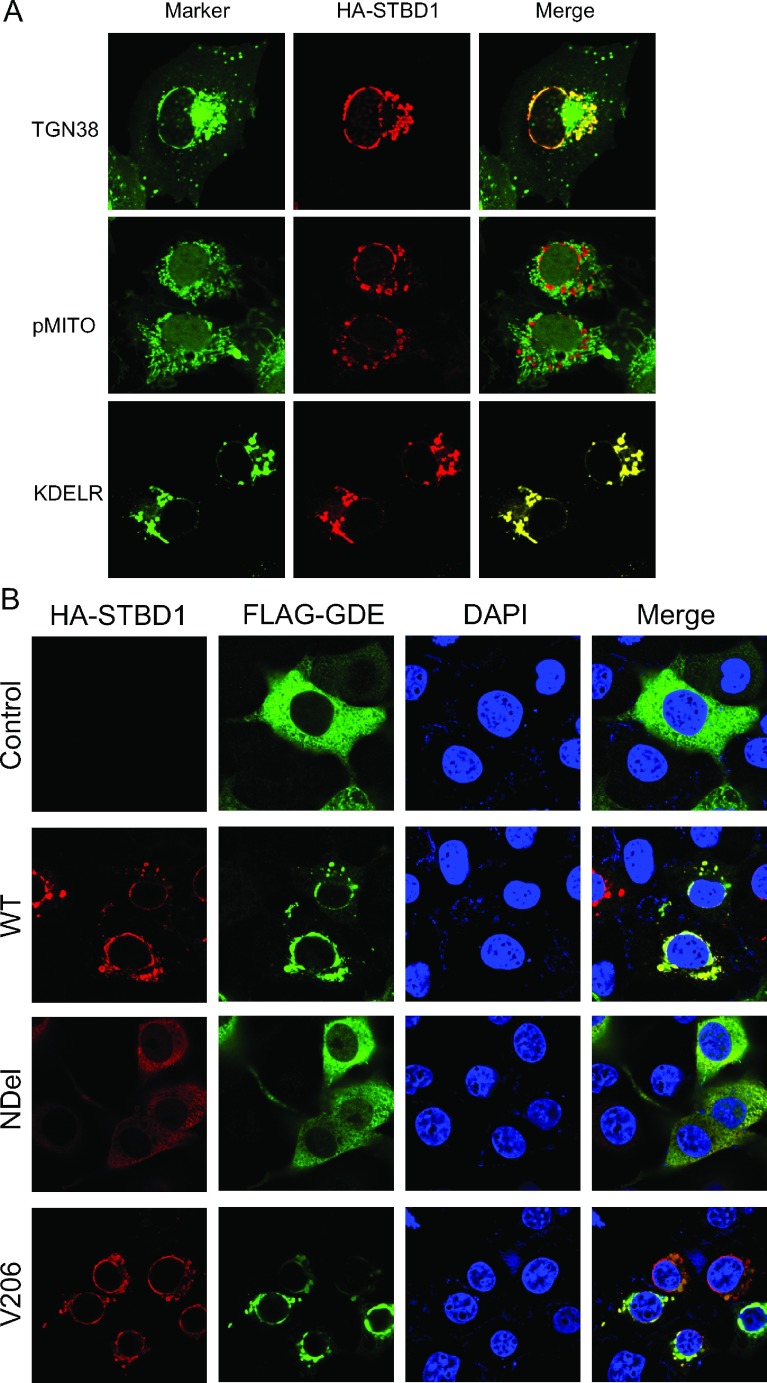
Expression of STBD1 causes relocalization of GDE to the ER/perinuclear region (**A**) COS cells were transfected with HA-STBD1 along with a GFP-tagged marker protein to decorate the subcellular compartment. Images were obtained by confocal microscopy. (**B**) Various HA-tagged STBD1 proteins were expressed in COS cells along with FLAG tagged GDE and analysed by confocal microscopy.

Although STBD1 possesses the ability to bind to glycogen, the subcellular localization of STBD1 is distinct from that of other glycogen-associated proteins. STBD1 is found at a perinuclear region that co-localizes with the ER (Figure 5A). To determine the exact subcellular compartment of STBD1 localization, we co-expressed GFP-tagged marker proteins to decorate the following subcellular compartments: the *trans*-Golgi network (GFP-TGN38), mitochondria (GFP-pMITO) and the ER (GFP-KDELR). As shown in Figure 5(A), STBD1 localizes to the ER, but not the mitochondria or the *trans*-Golgi network.

Since STBD1 interacts with several glycogen-associated proteins, we decided to investigate whether this association occurred at discrete locations within the cells. We have previously shown that GDE is located diffusely within liver cells [12,13]. However, co-expression of HA–STBD1 with FLAG–GDE caused a targeting of GDE to the ER compartment as well (Figure 5B). Similar results were observed with Laforin (results not shown).

Next, we tested whether expression of STBD1 mutant proteins could cause relocalization of GDE. The NDel mutant did not localize to ER/perinuclear region, suggesting that the N-terminal targets STBD1 to this localization. Furthermore, it did not cause relocalization of GDE. In contrast, the W203/V206 was able to exert this effect. Again, the W293 mutant was difficult to express, and did not cause relocalization to theER/perinuclear region (results not shown). Taken together, this suggests that STBD1 can form a complex with GDE via binding to its CBM20; and that this occurs at the ER/perinuclear region, where it is anchored by its N-terminal hydrophobic stretch.

### Increased binding of STBD1 to glycogen-associated proteins during glycogenolysis

**Figure 6:**
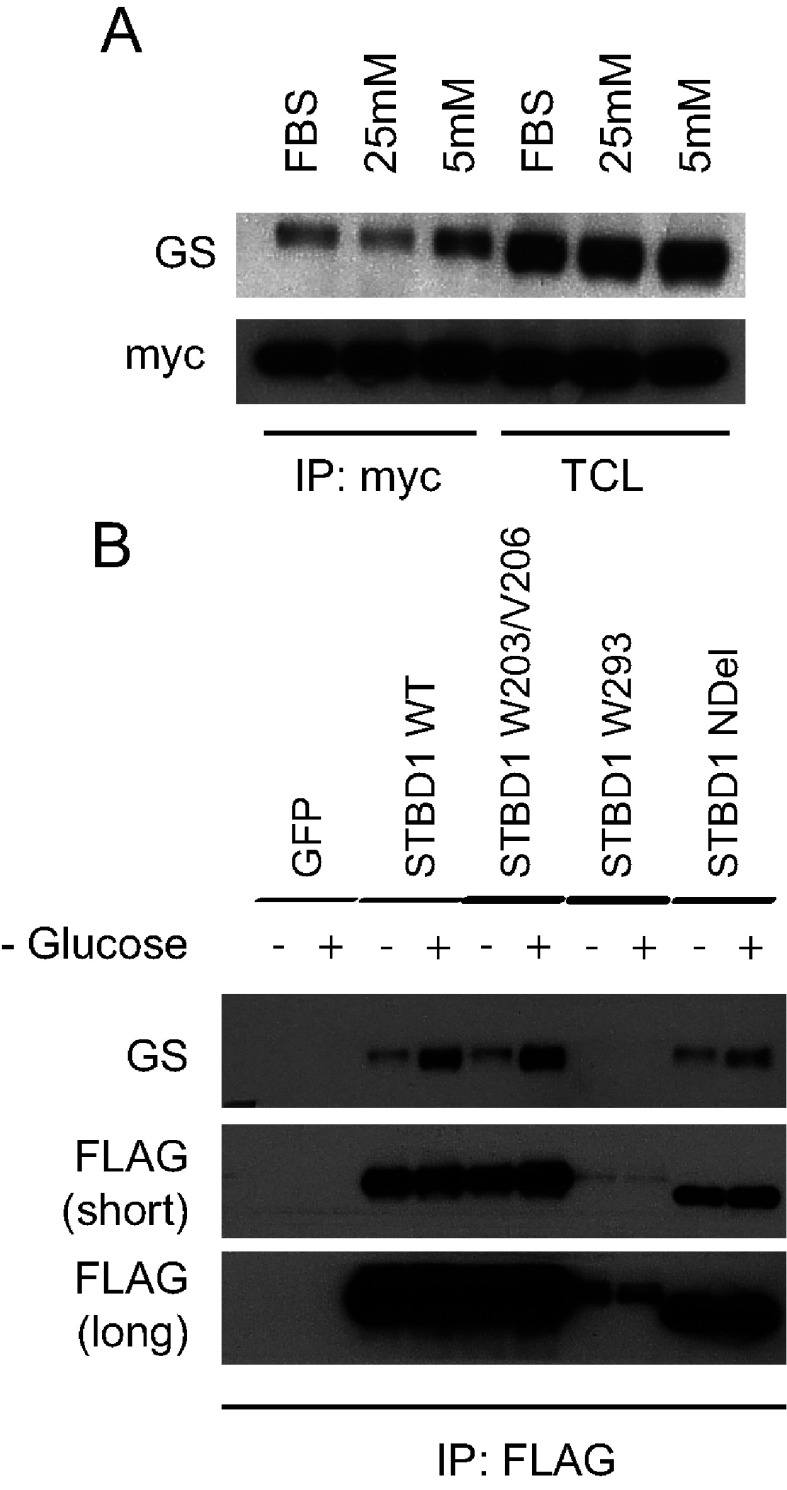
Increased binding of GS to STBD1 is regulated by glucose levels (**A**) HepG2 stable cell lines expressing myc-STBD1 were either cultured in regular media (FBS) or serum free media containing high (25 mM) or low (5 mM) glucose levels. Lysates were analysed for GS–STBD1 association by Western blotting. (**B**) HepG2 stable cell line expressing various FLAG-tagged STBD1 proteins were left untreated or subjected to glucose deprivation for 2 h. Lysates were analysed for GS–STBD1 association by Western blotting.

Up to this point, our experiments were performed in COS and Hela cells that have low glycogen levels. In cells that normally store glycogen, most proteins directly involved in its metabolism are also primarily associated with glycogen. We therefore hypothesized that during glycogenolysis, the associated proteins would be released and able to form complexes with STBD1. To test this possibility, we generated stable HepG2 liver cell lines of myc and FLAG-tagged STBD1 proteins or GFP as a control. The localization and stability of these proteins in HepG2 cells were similar to that in our Hela cell lines (results not shown). To determine whether binding of STBD1 to any of these glycogen-associated proteins is regulated, we cultured cells in regular media (with FBS) or serum free media with either 25 or 5 mM glucose and analysed the lysates for association of GS to STBD1 (Figure 6A). We tested for GS, since the antibody for this protein easily detected endogenous levels. In anti-FLAG immunoprecipitates, we observed increased binding of GS to STBD1 under low (5 mM) versus high (25 mM) glucose conditions.

Next, we assessed the binding during complete glucose deprivation. Cells were left untreated, or deprived of glucose for 2 h (Figure 6B). Similarly, during glucose deprivation, we observed an increase in GS–STBD1 association. This association was also observed with the NDel mutant and the W203/V206 mutant (Figure 6B). As a negative control, the GFP cell line did not show co-precipitation of GS in the immunoprecipitates. We also did not see an association with the W293 mutant, although its expression was significantly lower than the other proteins. These results suggest that there is an increased association between STBD1 and GS during glycogenolysis induced by glucose deprivation.

## DISCUSSION

STBD1 is a CBM20 containing protein implicated in both glycogen metabolism and autophagy. Previous studies have identified three regions/domains present on this protein. The CBM20 is able to bind to glycogen; however, its localization seems primarily dependent on its N-terminal hydrophobic stretch. Finally, recent studies also show that STBD1 can interact with proteins involved in autophagy [2,26]. Nevertheless, the cellular role of STBD1 has yet to be defined. Thus, the identification of additional binding partners, and determining the signals that regulate STBD1 may help our further understanding of this protein.

Based on our previous work with glycogen-binding domains [12,13], we decided to focus on the CBM20 motif of STBD1. Mutation of a key residue in this domain (W293) abrogated the ability of STBD1 to bind to amylose as expected. However, we also discovered that this caused STBD1 to be targeted to the proteasome for rapid degradation. This is similar to our previous data demonstrating that the glycogen-binding domain of GDE is important for protein stability [12,13]. Although evidence suggests that STBD1 participates in autophagy, our results show that inhibition of this pathway did not significantly alter the levels of STBD1, nor did it rescue the stability of the W293 mutant.

We also showed that both WT and the W293 mutant of STBD1 can undergo ubiquitination. This suggests that additional signals are involved in targeting it to the proteasome. Additionally, we also determined that the N-terminus of STBD1 is required for this process. This raises two possibilities. Either the N-terminus contains the ubquitination site(s) or the process requires STBD1 to be anchored to the ER/perinuclear region. Ongoing studies will hopefully answer these questions.

The CBM20 family, and eight other carbohydrate-binding module families, are often collectively classified as starch-binding domain containing proteins [3,4]. Starch is a major energy reserve for plants, and is chemically similar to glycogen, the equivalent storage form of glucose in animals [30]. However, their structural and physical properties are considerably different. Starch is composed of linear and branched glucose units; glycogen is not normally found as a linear molecule, and exhibits a higher degree of branching. Interestingly, *in vitro* experiments have shown that Laforin binds preferably to starch over glycogen [31].

Based on this information, one might expect that the CBM20 of STBD1 to behave similarly. However, our data, and those from others [1,2] strongly suggest that STBD1 localizes at the ER and lysosomes. Recent proteomic studies have indicated that STBD1 is a glycogen-associated protein in hepatocytes but not adipocytes [32,33]. This may be due to the fact that, in the hepatic glycogen proteome, endoplasmic proteins were also identified by the isolation procedure. Alternatively, this may reflect cell type differences, or the different pathways involved in glycogen metabolism.

Additionally, we have identified new interacting proteins of STBD1. Several glycogen-associated proteins such as GDE, Laforin and GS bind to STBD1, possibly via its CBM20. Laforin is a dual specificity phosphatase that is mutated in Lafora disease [16,17]. Several studies suggest that its primary role may involve the dephosphorylation of carbohydrates [34–36]. Additionally, Laforin has also been shown to bind to several glycogen-associated proteins, and has been proposed to target them for proteasomal mediated degradation [37,38]. It will thus be interesting to determine the effects of STBD1 on these functions of Laforin.

Concerning the STBD1 protein complexes, an obvious possibility is that these interactions are being bridged by glycogen. However, COS cells contain low levels of glycogen, and the high level of expression of overexpressed proteins makes it likely that the interaction is direct. More importantly, we observed that during glucose deprivation, when glycogenolysis occurs, the association of GS with STBD1 was enhanced. Therefore we propose that during glycogen breakdown, proteins such as GS, GDE or Laforin bind to STBD1. One intriguing possibility is that this binding acts as sensor to indicate when cells are low in glycogen levels.

Future efforts will be focused on determining the biological role(s) of STBD1. Recent work indicates that STBD1 might play a role in the degradation of glycogen by autophagy during metabolic stress in cardiovascular cells [7,8]. Those findings, further suggest that the expression of STBD1 is possibly regulated by various nutritional factors, such as fasting, insulin and cellular glycogen levels. Understanding how these conditions affect the STBD1 complexes (with proteins involved in autophagy and glycogen metabolism) should shed further light into its physiological roles.
